# Bandwidth-Aware Traffic Sensing in Vehicular Networks with Mobile Edge Computing

**DOI:** 10.3390/s19163547

**Published:** 2019-08-14

**Authors:** Kong Ye, Penglin Dai, Xiao Wu, Yan Ding, Huanlai Xing, Zhaofei Yu

**Affiliations:** 1 School of Information Science and Technology, Southwest Jiaotong University, Chengdu 611756, China; 2 College of Computer Science, Chongqing University, Chongqing 400040, China; 3 School of Electronics Engineering and Computer Science, Peking University, Beijing 100871, China

**Keywords:** traffic sensing, bandwidth-aware, vehicular networks, mobile edge computing, traffic state estimation

## Abstract

Traffic sensing is one of the promising applications to guarantee safe and efficient traffic systems in vehicular networks. However, due to the unique characteristics of vehicular networks, such as limited wireless bandwidth and dynamic mobility of vehicles, traffic sensing always faces high estimation error based on collected traffic data with missing elements and over-high communication cost between terminal users and central server. Hence, this paper investigates the traffic sensing system in vehicular networks with mobile edge computing (MEC), where each MEC server enables traffic data collection and recovery in its local server. On this basis, we formulate the bandwidth-constrained traffic sensing (BCTS) problem, aiming at minimizing the estimation error based on the collected traffic data. To tackle the BCTS problem, we first propose the bandwidth-aware data collection (BDC) algorithm to select the optimal uploaded traffic data by evaluating the priority of each road segment covered by the MEC server. Then, we propose the convex-based data recovery (CDR) algorithm to minimize estimation error by transforming the BCTS into an $l_{2}$-norm minimization problem. Last but not the least, we implement the simulation model and conduct performance evaluation. The comprehensive simulation results verify the superiority of the proposed algorithm.

## 1. Introduction

Traffic sensing is one of the promising applications in vehicular networks, which is critical to guarantee traffic system in safety and efficiency. Traditionally, vehicles driving on the road are regarded as mobile sensors [1,2] and periodically upload related traffic data to the access point (AP), such as cellular base station (BSs) and roadside unit (RSU), via wireless communication. Based on the collected traffic data, the traffic state of the whole urban area can be estimated and monitored, which is the fundamental to many emerging intelligent transportation systems, such as autonomous intersection control [3], traffic emergency warning [4], and adaptive path planning [5]. However, when vehicle density becomes intensive and network scale becomes large, periodically data uploading will impose high requirement on wireless bandwidth and computation resource. Furthermore, due to the limited wireless bandwidth and the highly dynamic vehicle mobility, traffic sensing always faces missing and inaccurate traffic data, which can badly degrade the system performance. Therefore, it is still not trivial to design an effective and efficient traffic sensing system in dynamic vehicular networks.

In the last decade, traffic sensing in vehicular networks has attracted great attention from the academic field. Traffic sensing system consists of two major mechanisms: data collection and data recovery. For data collection, some researchers [6,7] used the deployment of traffic camera distributed among the urban city to capture the traffic state of each road segment. However, this method requires the deployment of traffic cameras in the whole sensing area, which takes over-high deployment cost and can only be applied in some developed cities. Furthermore, some researchers [8] proposed a traffic sensing architecture, where vehicles equipped with types of sensing devices are regarded as mobile sensors and collect traffic data along their driving path. In particular, [9,10] used vehicles as road probes for collecting traffic data independently, which compresses the collected data based on compressive sensing theory and uploads them in every time period for bandwidth saving. However, due to the random mobility of vehicles, it is difficult to select the optimal probing vehicles and control the distribution of covered road segments. Then, some researchers [11,12,13] proposed a cooperative data selection strategy, where traffic data of multiple vehicles is aggregated via multi-hop vehicle-to-vehicle communication and finally uploaded by a seed vehicle. In particular, coding technique is used during data transmission for reducing the amount of data transmission. Though covering more sensing area, this method takes long transmission delay and tolerates data loss during multi-hop Vehicle-to-Vehicle (V2V) communications. On the other hand, compressive sensing technique is commonly adopted in data recovery of traffic sensing in vehicular networks. Specifically, the insight principle [14,15] is to construct a sparse matrix by finding out temporal and spatial correlation between missing data and available data and recover the missing data by solving the norm minimization problem. However, currently, these solutions suffer from high estimation error when the missing data increases. Furthermore, when the network scale of sensing area becomes large, the communication and computation cost also increase dramatically.

Recently, mobile edge computing (MEC) [16,17] has become one of the most promising paradigms by offloading resources at the network edge, especially in vehicular networks. In particular, each MEC server installed along the roadside, owns computation and communication capabilities, which can undertake local service computing for vehicles via wireless communication without the help of remote cloud. For traffic sensing, the MEC service architecture provide the chance of flexible data collection strategy and efficient data recovery at local server, which alleviates much pressure on the central cloud and backbone networks. Specifically, the MEC server can control the data selection strategy, which provides possibility of flexible data selection among mobile vehicles within limited bandwidth constraint. Furthermore, the data recovery is implemented at the MEC server, which brings computation offloading at network edge and reduces extra transmission cost between terminal users and cloud servers.

Based on the above motivation, this paper investigates a traffic sensing architecture in MEC-based vehicular networks. Specifically, each MEC server is distributed along the roadside and responsible for monitoring the road-level traffic state in its service range. In particular, traffic state of each road segment covered by the MEC server, which is defined as the section of segment between two adjacent intersection point, is monitored. In such an architecture, the large-scale sensing area can be divided into multiple sub-areas and the traffic sensing of each sub-area is implemented at each MEC server independently. However, it is still challenging to apply such a service architecture for traffic sensing in vehicular networks. The first challenge is that how to select the optimal partial traffic data under limited wireless bandwidth. In particular, the traffic features of each road segment differ from each other, including vehicle density and average speed, and may change dynamically over time. Unbalanced data collection among multiple road segments may also degrade estimation accuracy. Then, the second is that how to recover or estimate the missing elements based on the collected traffic data, especially when the traffic data of some road segments is totally missing. The third is that the optimization model considering both traffic data collection and recovery is formulated as a non-linear mixed integer programming model, which is typically NP-hard and cannot be directly solved in an efficient way.

Based on the above observation, this paper is dedicated to solving the above-mentioned problem. As far as we know, it is the first work to investigate the traffic sensing in the MEC-based vehicular networks, including both data collection and recovery, by considering the heterogeneous network characteristics of MEC servers. The contributions of this paper are outlined as follows.

- First, we propose an MEC-based service architecture for traffic sensing system in vehicular networks, where each MEC server is responsible for managing the data upload of vehicles in its service range and estimating the traffic state based on collected data set.
- Second, we formulate the problem of bandwidth-constrained traffic sensing (BCTS) by synthesizing the heterogeneous capacities of MEC servers and dynamic mobility features of vehicles, which aims at minimizing the estimation error between the original traffic state and the estimated traffic state.
- Third, to tackle the BCTS problem, we propose two algorithms for data collection and data recovery, respectively. First we propose a bandwidth-aware data collection (BDC) for selecting the optimal upload data set by adaptively capturing the temporal and spatial correlation of traffic data base on historical data set. Furthermore, we propose a convex-based data recovery (CDR) algorithm to estimate the full traffic state in the whole sensing area by transforming the BCTS problem into a norm minimization problem.
- Fourth, we implement the system model by integrating the realistic traffic data with real-world map, as well as the proposed algorithm. The comprehensive simulation result shows the superiority of the proposed algorithms compared with two competitive algorithms under various circumstances.

The rest of this paper is organized as follows. Section 2 reviews the related work. Section 3 presents the MEC-based service architecture. Section 4 formulates the BCTS problem. In Section 5, we propose the data collection and recovery algorithms. In Section 6, we build the simulation model and evaluate the algorithm performance. Finally, Section 7 concludes this paper and discusses the future work.

## 2. Related Work

The traffic sensing system is supposed to consist of two major mechanism: data collection and data recovery. We will introduce the related work in the two aspects. First, data collection of traffic sensing in vehicular network is generally classified into two categories, non-cooperative data collection and cooperative data collection, respectively. Specifically, [9,18] are non-cooperative strategy, in which each vehicle is regarded as an individual road probe for independent data acquisition and then compressed the collected data by itself for data upload in every fixed time period. Lin et al. [9] designed a real-time compressive sensing (CS) approach, which allows vehicles to collect and compress data in real time and can recover the original data accurately and efficiently. Alasmary et al. [18] identified the sparsity of the vehicle tracking information and proposed a compressed sensing information recovery scheme. The proposed scheme reduces the amount of data exchanged due to vehicle tracking packets while providing a robust information reception at the receivers. However, this type of data processing imposes high requirement of computation on vehicles. On the other hand, for cooperative data collection, the traffic data of multiple vehicles is aggregated via multi-hop V2V transmission [19,20,21]. Liu et al. [20] proposed a novel scheme called compressive sensing-based data collection (CS-DC), which can efficiently collect spatially correlated data in vehicular networks. In particular, CS-DC can efficiently reduce communication overhead with low computation and less communication control. Wang et al. [19] proposed a compressive sensing-based approach (CSM) to monitor in vehicular networks. In particular, the CSM can make a balanced tradeoff between communication cost and estimation accuracy and guarantee estimation accuracy over the highly dynamic network. However, when the vehicular topology changes dramatically, cooperative data collection may result in long transmission cost and data loss during multi-hop V2V communication. Except for the vehicle-centric data collection method, the data collection in vehicular networks can also rely on fixed devices such as APs or RSU [9,22,23]. The APs and RSUs can provide more reliable data collection for specific area since they are fixed. However, when the wireless bandwidth becomes bottlenecks, especially in the case of high vehicle density, they still lose some traffic data, which also result in inaccuracy traffic evaluation. In this paper, we will investigate the efficient data collection strategy for the MEC server, which aims at capturing the major traffic features when wireless bandwidth is not sufficient.

Data recovery in traffic sensing is used for estimating the erroneous or missing data occurred during the data collection. Xu et al. [24] analyzed the compressibility of road state data and proposed a method of road traffic state estimation based on compressed sensing. Chen et al. [25] developed a novel decomposition technique to accurately decompose a network matrix into a low-rank matrix, a sparse anomaly matrix, an error matrix, and a small noise matrix, which is used for removing noisy data. Li et al. [26] proposed a new approach based on compressive sensing to large-scale traffic sensing in urban areas, which mines the extensive real trace datasets of taxis in an urban environment with principal component analysis and reveals the existence of hidden structures with sensory traffic data that underpins the compressive sensing approach. However, these solutions cannot guarantee the estimation accuracy when the portion of missing data is high. Based on the above observation, this work innovatively proposes a traffic sensing strategy, which consists of both bandwidth-area data collection and convex-based data recovery strategy, which can intelligently select the traffic data with the most dominant traffic features in a bandwidth-constrained scenario and achieve low estimation error based on a dedicated designed gradient descent method.

## 3. System Model

**Figure 1:**
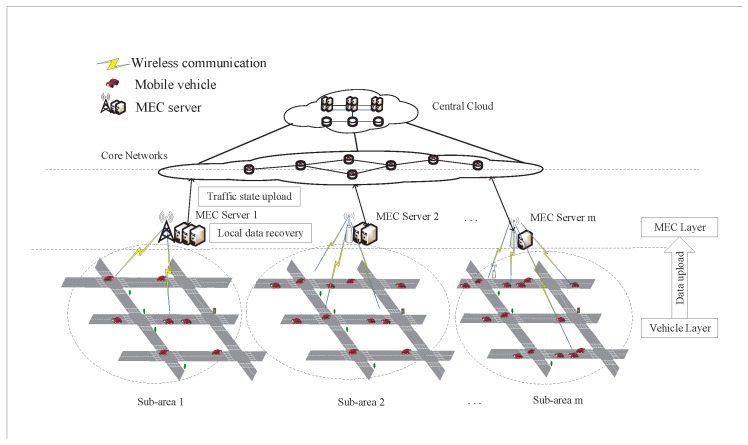
Service Architecture of Traffic monitoring in MEC-based Vehicular Networks.

In this section, we present the service architecture for traffic sensing system in MEC-based vehicular networks. Typically, as shown in Figure 1, the service architecture consists of three layers: vehicle layer, MEC layer, and cloud layer. In the vehicle layer, the whole traffic sensing area is divided into sub-areas and each sub-area is exactly covered and monitored by a MEC server. In particular, there exists no overlap between different sub-areas. The traffic state of each sub-area is evaluated in road segment level. Specifically, the road segment is defined as the road section between two adjacent intersections. The traffic state of each road segment is determined by the mobility features of dwelling vehicles. In the MEC layer, the MEC server is responsible for offloading traffic sensing task in its local server, which includes data collection and data recovery. Accordingly, the MEC server owns a wireless AP and a computation server. As shown in Figure 1, the MEC server can have heterogeneous wireless interfaces, such as RSU with Dedicated Short Range Communication (DSRC), BS with (3G, 4G and 5G) cellular interface, which is commonly adopted in the relevant literature [27]. Furthermore, the wireless bandwidth of a wireless AP is defined as the maximum amount of data can be uploaded per time unit, which limits the amount of uploaded vehicle data in the sub-area. The heterogeneity of the wireless APs is characterized by different wireless bandwidths. Then, the computation server is responsible for recovering the realistic traffic state by processing the collected data set with missing data. In cloud layer, the central cloud is responsible for monitoring the traffic states in the system level by collecting the estimated traffic states from the MEC layer via wired connection. This paper assumes that the wired bandwidth is sufficient to support data exchange between the MEC layer to the cloud layer. Such an MEC-based architecture is also adopted for other data services in vehicular networks in the relevant literature [17,28]. In fact, the reliability issue is not considered in this paper. Similar to [29], this paper assumes that the proposed service architecture is reliable to support the traffic sensing solution implemented at individual MEC server.

**Figure 2:**
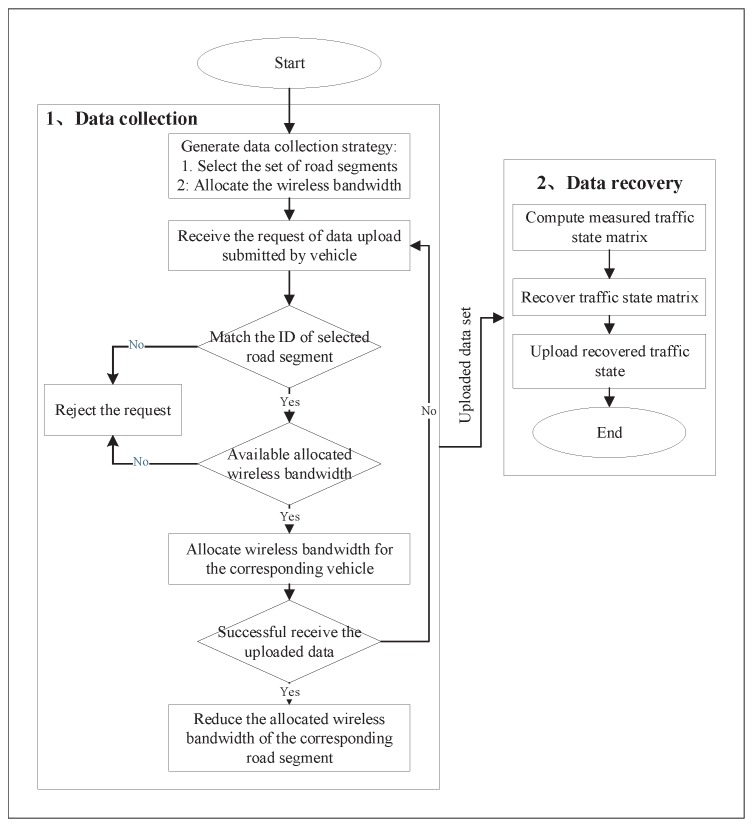
Data Processing of the MEC Server.

The detail processing procedure of the MEC server consists of two components: data collection and data recovery, which is shown in Figure 2. On one hand, the MEC server makes data collection strategy, which includes the collected road segment selection and the wireless bandwidth allocation. In this paper, we assume that the data collection strategy is determined in offline phase based on historical collected data set. Second, the MEC server can receive the data upload request by overhearing the beacon message broadcast by the vehicle. The beacon message includes the required bandwidth as well as the ID of road segment associated with the vehicle. It is reasonable that the vehicle can sense the ID of dwelling road segment based on the GPS data. Third, the MEC server will check whether the ID of road segment is selected or not. If the ID is not matched, the MEC server will reject the request. Otherwise, it goes to step four. Fourth, the MEC server further checks that whether the required bandwidth can be satisfied or not. If the condition is not satisfied, the MEC server will reject the request. Otherwise, the MEC server will allocate the bandwidth for the vehicle. Fifth, the MEC server is ready for receiving the uploaded vehicle data. If the data is uploaded successfully, the MEC server will update the allocation bandwidth for the corresponding road segment. Otherwise, the allocation bandwidth will not be updated. During the data collection procedure, the MEC server only cares the amount of uploaded data in road segment level without managing the mobility of individual vehicle. On the other hand, for data recovery, the MEC server must estimate the realistic traffic state based on the collected traffic data of the sub-area. First, the MEC server computes the measured traffic state of each road segment. Furthermore, by designing the dedicated data recovery mechanism, the MEC server estimates the realistic traffic state based on the measured traffic state.

## 4. Problem Formulation

### 4.1. Preliminary

Let *M* be the set of MEC servers. Each MEC server m∈M is characterized by three-tuple $\left(p_{m},R_{m},W_{m}\right)$. Specifically, $p_{m}$ and $R_{m}$ represents the location and the set of covered road segment of *m*, respectively. In particular, $W_{m}$ is the maximum bandwidth owned by *m*, which is defined as the maximum amount of data can be uploaded per time unit in its service range. Therefore, for each vehicle *v*, the uploaded vehicle data $d_{v}$ is characterized by four-tuple $\left(id_{v},p_{v},v_{v},t_{v}\right)$, which indicates the identity, the location, the velocity and the time stamp, respectively.

### 4.2. Bandwidth-Constrained Traffic Sensing

In this section, we formulate the problem of Bandwidth-constrained traffic sensing (BCTS) in detail.

First, we derive the formulation for evaluating the traffic state of each road segment $r_{i}\in R_{m},m=1,2,3,\ldots ,M$. Due to different sampling rates of vehicles, it is difficult to track the traffic state with a consistent time stamp. Therefore, we divide the time interval into multiple time periods with fixed length and evaluate the traffic state at the scale of time period. Therefore, we define the set $S^{o}\left(i,j\right)$ as the realistic traffic data set of vehicles on road segment $r_{i}\in R_{m}$ at each time period $t_{j}$, which is defined as follows.

(1) $$S^{o}\left(i,j\right)=\{d_{v}\;|\;\;||p_{v}-p_{i}\left|\right|\leq \rho_{1},||t_{v}-t_{j}\left|\right|\leq \rho_{2}\}$$

where $\rho_{1}$ and $\rho_{2}$ are two predefined thresholds. Based on Equation (1), the realistic traffic state of each road segment $r_{i}\in R_{m}$ at each time period $t_{j}$, denoted by $x_{ij}^{o}$, is defined as the average speed of $d_{v}\in S^{o}\left(i,j\right)$, which is formulated as follows.

(2) $$x_{ij}^{o}=\frac{\sum_{\forall d_{v}\in S^{o}\left(i,j\right)}v_{v}}{\left|\right|S^{o}\left(i,j\right)\left|\right|}$$

Therefore, we can acquire the realistic traffic state matrices $X_{m}^{o}$, ∀m∈M, which is expressed as follows.

(3) $$\left[\begin{array}{cccc} x_{11}^{o} & \ldots & \ldots & x_{1T}^{o}\\ x_{21}^{o} & \ldots & \ldots & x_{2T}^{o}\\ \ldots & \ldots & \ldots & \ldots \\ x_{R_{m}1}^{o} & \ldots & \ldots & x_{R_{m}T}^{o} \end{array}\right]$$

when the wireless bandwidth is sufficient, all the traffic data can be uploaded, then $X_{m}^{o},m=1,2,\ldots ,M$ can be easily computed by Equation (2).

However, in fact, the amount of uploaded data is always constrained by the limited wireless bandwidth. Then, $\left|\right|I_{m}\left|\right|\times \left||T|\right|$ indicator matrix of MEC server *m*, where each $b_{m}\left(i,j\right)\in B_{m}$ indicates whether road segment $r_{i}$ at $t_{j}$ is selected for traffic data collection or not, which is defined as follows.

(4) $$b_{m}\left(i,j\right)=\left\{\begin{array}{cc} 1, & if\;(r_{i},t_{j})\;is\;selected\\ 0, & otherwise \end{array}\right.$$

Furthermore, $S^{c}\left(i,j\right)$ denotes the collected traffic data set of road segment $r_{i}\in R_{m}$ at each time period $t_{j}$. Therefore, $S^{c}\left(i,j\right)$ is the subset of $S^{o}\left(i,j\right)$, i.e., $S^{c}\left(i,j\right)\subset S^{o}\left(i,j\right)$. Therefore, the total amount of collected data set of m∈M at each time period $t_{j}$ cannot exceed the communication capacity of *m* (denoted by $W_{m}$), which is formulated as follows.

(5) $$\sum_{i=1}^{R_{m}}\sum_{j=1}^{T}b_{m}\left(i,j\right)\cdot \left|\right|S^{c}\left(i,j\right)\left|\right|\leq W_{m},\forall m\in M$$

Similarly, we can compute the collected traffic state matrix $X_{m}^{c},m=1,2,\ldots ,M$ based on Equation (2). However, there exist missing elements, $X_{m}^{c}$ always deviates greatly from the realistic traffic state matrix $X_{m}^{o}$. Therefore, we must derive the estimated traffic matrix ${\hat{X}}_{m}$ based on $X_{m}^{c}$. The estimation function of mapping from $X_{m}^{c}$ to ${\hat{X}}_{m}$ is generalized as follows.

(6) $${\hat{X}}_{m}=f\left(X_{m}^{c}\right)$$

Based on the above analysis, given realistic traffic data set $S^{o}\left(i,j\right)$, the optimization problem is formulated as follows.

(7) $$\begin{array}{c} min\sum_{\forall m\in M}\left|\right|X_{m}^{o}-{\hat{X}}_{m}{\left|\right|}_{2}\\ S.T.\sum_{i=1}^{R_{m}}\sum_{j=1}^{T}\left|\right|S^{c}\left(i,j\right)\left|\right|\cdot b_{m}\left(i,j\right)\leq W_{m},\forall m\in M\\ \;\;{\hat{X}}_{m}=f\left(X_{m}^{c}\right),\forall m\in M\\ \;\;S^{c}\left(i,j\right)\subset S^{o}\left(i,j\right),b_{m}\left(i,j\right)\in \left\{0,1\right\},\forall r_{i}\in R_{m},m\in M,j=1,2,\ldots ,T \end{array}$$

As we can see, it is a non-linear mixed integer programming problem, which is a known Non-deterministic Polynomial (NP)-hard problem. Furthermore, since the original matrices $X_{m}$, m=1,2,…,M cannot be known in advance and the estimation function *f* is not determined , the optimization model cannot be directly solved in the form of Equation (7). non-deterministic polynomial

## 5. Algorithm Design

In this section, we propose two algorithms for data collection and recovery, respectively. These two algorithms are based on singular value decomposition (SVD). Hence, before elaboration, we first introduce the principle of singular value decomposition.

### 5.1. Compressive Sensing-Based Singular Value Decomposition

Singular Value Decomposition [30] is a basic tool for matrix decomposition, which can decompose a m×n matrix *X* into the multiplication of three matrices.

(8) $$X=U\Delta V^{T}$$

where *U* is a m×m unitary matrix (i.e., $UU^{T}=U^{T}U=I$) and *V* is an n×n unitary matrix (i.e., $VV^{T}=V^{T}V=I$), and Δ is an m×n diagonal matrix containing the singular values $\sigma_{i}$ of *X*, and $\sigma_{i+1}\leq \sigma_{i}$. The rank of a matrix is the number of rows or columns that are linearly independent, and its value is equal to the number of non-zero singular values.

For simplicity, let $P=U\Delta^{1/2}$ and $Q=V^{T}\Delta^{1/2}$, then the singular value decomposition of *X* (defined in Equation (8)) can be transformed into the product of two matrices *P* and *Q*, formulated as follows.

(9) $$X=U\Delta V=PQ^{T}$$

furthermore, we denote the ith row vector of *P* and the jth row vector of *Q* as $p_{i}$ and $q_{j}$, respectively. Then, the element $x_{ij}\in X$ can be represented by $p_{i}{q_{j}}^{T}$.

### 5.2. Bandwidth-Aware Data Collection Algorithm

In this section, we propose the bandwidth-aware data collection (BDC) algorithm. The principle of BDC is to evaluate the temporal and spatial correlation of road segments based on historical collected data set. Given the historical data set $S^{pre}\left(i,j\right)$, $\forall r_{i}\in R_{m},m\in M,j=1,2,\ldots ,T$, then $X_{m}^{pre}$ can be computed based on Equation (2).

Based on SVD method (defined in Equation (8)), $X_{m}^{pre}$ can be decomposed as follows.

(10) $$X_{m}^{pre}=U_{R_{m}\times R_{m}}^{pre}\Delta_{R_{m}\times T}^{pre}V_{T\times T}^{pre}$$

where $\sigma_{i+1}$ of Δ is supposed to contain the hidden structure of the traffic state. The magnitude of the hidden structure is supposed to be proportional to the value of $\sigma_{i+1}$. We maintain the *K* largest singular values of Δ as well as the corresponding *K* row vectors of $U^{pre}$ and *K* row vectors of $V^{pre}$. After that, we can acquire three matrices $U_{R_{m}\times K}^{pre}$ and $\Delta_{K\times K}^{pre}$ and $V_{K\times T}^{pre}$. Therefore, the traffic state matrix can be approximated as follows.

(11) $$X_{m}^{pre^{\prime }}=U_{R_{m}\times K}^{pre}\Delta_{K\times K}^{pre}V_{K\times T}^{pre}=P^{pre^{\prime }}Q^{pre^{\prime }}$$

where $P^{pre^{\prime }}=U_{R_{m}\times K}^{pre}\Delta_{K\times K}^{1/2}$ and $Q^{pre^{\prime }}=\Delta_{K\times K}^{1/2}V_{K\times T}$.

Based on Equation (11), this paper evaluates the correlation magnitude of each road segment $r_{i}\in R_{m}$ at each time period $t_{j}$. For each element $x_{ij}^{pre}$ in $X^{pre}$, we replace $x_{ij}^{pre}$ by 0 and compute estimation of $x_{ij}^{pre^{\prime }}$ as $p_{i}^{pre^{\prime }}q_{j}^{pre^{\prime }}$. Then, we define the priority of the element as follows.

(12) $$G_{m}\left(r_{i},t_{j}\right)=\frac{|x_{ij}-x_{ij}^{{}^{\prime }}|}{x_{ij}}=\frac{|x_{ij}-p_{i}^{{}^{\prime }}{q_{j}^{{}^{\prime }}}^{T}|}{x_{ij}}$$

**Figure 3:**
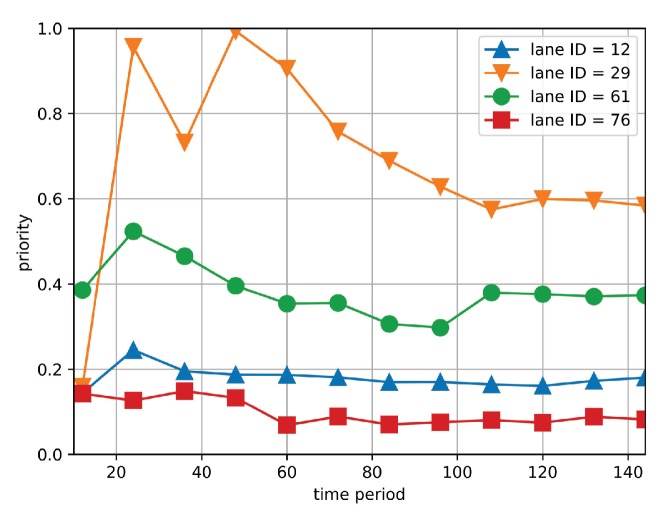
The priority of multiple road segments under different time periods.

**Figure 4:**
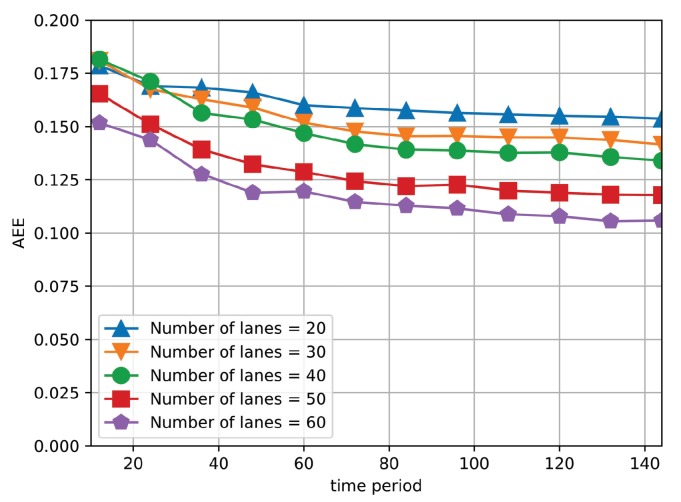
The AEE of four sets of road segments under different time periods.

Based on Equation (12), high value of $G_{m}\left(r_{i},t_{j}\right)$ indicates that the difference between $x_{ij}^{prev}$ and $x_{ij}^{prev^{\prime }}$ is large and therefore the traffic data of road segment $r_{i}$ at $t_{j}$ has high priority to be collected. For validation, we compare the priority of four different road segments, respectively. Specifically, we recover the value of $x_{ij}^{{}^{\prime }}$ under the condition of missing the information of road segment $r_{i}$ and compute $G_{m}\left(r_{i},t_{j}\right)$ based on Equation (12). The related setup is referred to Section 6. Figure 3 shows the priority of four missing road segments under different time periods. It is observed that the trend of the four curves differs from each other, which reveals different correlation magnitude of elements in $X^{pre}$. For instance, in general, the priority of road segment 29 maintains at the highest level among the four curves, which indicates that the traffic data of road segment 29 has higher priority to be collected compared with other road segments. Furthermore, we define average estimation error (AEE) as the difference between the estimated and original traffic matrix, which is formulated in Equation (17). The detailed definition of AEE is referred to in Section 6. Figure 4 shows the AEE of four sets of road segments with varying *k* largest priority, where *k* changes from 20 to 60. When *k* increases, the AEE decreases. It is because that more road segments provide larger amount of collected traffic data. However, it is observed that even *k* is small (i.e., k=20), the priority of the curve still maintains at a preferable level (i.e., 0.17), which gives an inspiration that low estimation error can still be achieved based on the traffic data with high priority even when the wireless bandwidth is constrained.

Therefore, the procedure of the BDC algorithm is described as follows. First, for each $r_{i}\in R_{m}$ at each $t_{j}$, the priority of each $r_{i}$ at each $t_{j}$ is computed based on Equation (12). The detailed procedure is shown in lines 1∼10 of Algorithm 1. Second, the priority of road segments at each time period $t_{j}$ is sorted in the descending order and the indexes are stored in the index matrix *I*. The detailed procedure is shown in in lines 11∼17 of Algorithm 1 . Third, for each time period $t_{j}$, the collected data set $S_{m}^{c}\left(i,j\right)$ of each road segment $r_{i}$ is determined iteratively in the order of I(:,j). In particular, let $l_{upload}$ and $T_{left}$ denote the maximum number of elements in $S_{m}^{c}\left(i,j\right)$ and the remaining communication capability, then the number of uploaded data item for $r_{i}$ in $t_{j}$ is determined by $L=min(T_{left},l_{upload})$.For each road segment $r_{i}$ is selected, where i∈I(:,j), we set $b_{m}\left(i,j\right)$ to 1 and then randomly select *L* elements from $S^{o}\left(i,j\right)$ as $S_{m}^{c}\left(i,j\right)$. Accordingly, $x_{ij}^{c}$ can be computed based on $S_{m}^{c}\left(i,j\right)$ by Equation (2). The detailed procedure is shown in lines 18∼31 of Algorithm 1. It is observed that the procedure of Step 1 of BDC algorithm can be implemented in offline phase since priority of the road segment is determined based on historical data set. Furthermore, the time complexity of Step 2 is O(T), where *T* is the scheduling period. When the scheduling period *T* is considered to be a constant, then the time complexity of BDC can be regarded as a constant, which indicates that BDC can be implemented at each MEC server in an efficient way.

**Algorithm 1** The Bandwidth-aware Data Collection (BDC) Algorithm**Input:** Historical data set $S^{prev}\left(i,j\right)$,$\forall r_{i}\in R_{m},m\in M,j=1,2,...,T$ **Output:** The collected traffic state matrix $X_{m}$ and indicator matrix $B_{m}$, ∀m∈M **Step 1:** determine the selection priority of the road segments (Offline Phase)
  1:Compute $X_{m}^{prev}$ based on Equation (2), ∀m∈M  2:Set priority matrix $G_{m}={\left[0\right]}_{R_{m}\times T}$  3:**for** each $r_{i}\in R_{m}$ **do**  4: **for** *j* from 1 to *T* **do**  5:  $X_{m}^{prev^{\prime }}\leftarrow X_{m}^{prev}$  6:  $x_{ij}^{prev^{\prime }}\leftarrow 0$  7:  Decompose $X_{m}^{prev^{\prime }}$ as $P_{m}^{prev^{\prime }}$ and $Q_{m}^{prev^{\prime }}$ based on Equation (11)  8:  $x_{ij}^{prev^{\prime }}\leftarrow p_{i}^{prev^{\prime }}q_{j}^{prev^{\prime }}$  9:  Compute $G_{m}\left(r_{i},t_{j}\right)$ based on Equation (12)10: **end for**11:**end for**12:Set index matrix I=∅13:**for***j* from 1 to *T* **do**14: tmp← the *jth* column row vector of $G_{m}$15: Sort elements of tmp in descending order16: tmp_index← the index vector of sorted elements in tmp17: I←[I,tmp_index]18:**end for**
**Step 2:** allocate wireless bandwidth and collect vehicle data (Online Phase)
19:**for***j* from 1 to *T* **do**20: $T_{left}=W_{m}$21: **for** each i∈I(:,j) **do**22:  Set $b_{m}\left(i,j\right)=1$23:  $L=min(T_{left},l_{upload})$24:  **if** L>0 **then**25:   $S^{c}\left(i,j\right)\leftarrow$ randomly select *L* elements from $S^{o}\left(i,j\right)$26:   Compute $x_{ij}^{c}$ by Equation (2)27:  **else**28:   break29:  **end if**30:  $T_{left}=T_{left}-L$31: **end for**32:**end for**33:Output $X_{m}^{c}$ and $B_{m}$


### 5.3. Convex-Based Data Recovery Algorithm

In this section, we propose the convex-based data recovery (CDR) algorithm to derive the estimated traffic state matrix ${\hat{X}}_{m}$ based on the collected traffic state matrix $X_{m}^{c}$. Since the objective function (defined in Equation (7)) cannot be directly computed, we transform the optimization model as follows.

(13) $$\begin{array}{c} \min\;rank\left(\hat{P}{\hat{Q}}^{T}\right)\\ s.t.\;B_{m}.\times \left(\hat{P}{\hat{Q}}^{T}\right)=X_{m}^{c} \end{array}$$

however, it is still difficult to solve the optimization model defined in Equation (13), since rank minimization of matrix is a non-convex problem. However, when PQ satisfies the restricted isometry property (RIP) condition [19,31], Equation (13) can be transformed into a $l_{2}$-norm minimization problem.

(14) $$min\;\lambda (||\hat{P}{\left|\right|}^{2}+\left|\right|\hat{Q}{\left|\right|}^{2})+||B_{m}.\times \left(\hat{P}\hat{Q}\right)-X_{m}^{c}{\left|\right|}^{2}$$

however, when the matrix satisfies the RIP condition, the estimated value ${\hat{x}}_{ij}$ is supposed to be relatively smaller than $x_{ij}^{o}$. Therefore, in this paper, we add a complementary factor $C_{m}\Omega_{m}$ to each element ${\hat{x}}_{ij}$. Specifically, $\Omega_{m}$ is formulated as follows.

(15) $$\Omega_{m}=\frac{\sum_{\forall x_{ij}^{c}\in X_{m}^{c},x_{ij}^{c}\neq 0}x_{ij}^{c}}{\sum_{\forall x_{ij}^{c}\in X_{m}^{c},x_{ij}^{c}\neq 0}1}$$

then, for each m∈M, $C_{m}$ is defined as the ratio of the number of collected road segments and the total number of road segments, which is defined as

(16) $$C_{m}=\frac{\sum_{\forall x_{ij}^{c}\in X_{m}^{c},x_{ij}^{c}=0}1}{T\times R_{m}}$$

The procedure of the CDR algorithm consists of three steps, shown in Algorithm 2, which is illustrated as follows. In the first step, we initialize the related parameters, including the $C_{m}$, $\Omega_{m}$, and two matrices $\hat{P}$ and $\hat{Q}$, which is shown in lines 1∼6 in Algorithm 2. The rank of $\hat{P}$ and $\hat{Q}$ is determined by rank factors *r*. Second, we perform the gradient descend method to achieve the minimum value of Equation (14) in an iterative way. During the iteration, the regularization coefficient λ is used for preventing the over-fitting of the estimated data and the learning rating lr is used for controlling the process speed of estimating the traffic state matrix data. The stop condition is that the difference between pre_e and *e* is smaller than a predefined threshold $\rho_{3}$. After that, we can acquire the estimated traffic matrix ${\hat{X}}_{m}$. Third, for each element ${\hat{x}}_{ij}\in {\hat{X}}_{m}$, we add the complementary factor $C_{m}\Omega_{m}$ to ${\hat{x}}_{ij}$, i.e., $\hat{x}{}_{i,j}=\hat{x}{}_{i,j}+\Omega_{m}\cdot C_{m}$. Then, we can acquire the final estimated traffic state matrix ${\hat{X}}_{m}$ for MEC server m∈M. The effect of three parameters related to Algorithm 2 will be investigated in detail in Section 6. Additionally, it is observed that the CDR algorithm can be implemented and performed at each individual MEC server independently. It is observed that the time complexity of Algorithm 2 is dominated by the procedure in the while loop, shown in lines 7∼21, whose time complexity is $O(t||R_{m}||\cdot ||T||)$. Specifically, *t*, $\left|\right|R_{m}\left|\right|$ and ||T|| are the iteration number, the number of covered road segments and the scheduling period. In fact, it is verified in Section 6 that the CDR can converge when the iteration number *t* is small. Therefore, the time complexity of Algorithm 2 is linear to $\left|\right|R_{m}\left|\right|$, i.e., linear to the number of covered road segments.

**Algorithm 2** The Convex-based Data Recovery (CDR) Algorithm**Input:** Collected traffic state matrix $X_{m}^{c}$, indication matrix $B_{m}$, regularization coefficient λ, rank factor *r*, and learning rating lr **Output:** Estimation matrix $\hat{X}{}_{m}$
  1:$\hat{P}\leftarrow$ a m×r matrix generated by standard normal distribution  2:$\hat{Q}\leftarrow$ a m×r matrix generated by standard normal distribution  3:Compute $\Omega_{m}$ based on Equation (15)  4:Compute $C_{m}$ based on Equation (16)  5:Initialize e_diff=2  6:pre_e=0  7:**while**$e\_diff>\rho_{3}$**do**  8: **for** *i* from 1 to $R_{m}$ **do**  9:  **for** *j* from 1 to *T* **do**10:   ${\hat{q}}_{i}={\hat{q}}_{i}+lr\cdot \left(e\cdot {\hat{p}}_{j}-\lambda {\hat{q}}_{i}\right)$11:   ${\hat{p}}_{j}={\hat{p}}_{j}+lr\cdot \left(e\cdot {\hat{q}}_{i}-\lambda {\hat{p}}_{j}\right)$12:  **end for**13: **end for**14: $e=\lambda (\|\hat{P}{\|}^{2}+\|\hat{Q}{\|}^{2})+\|B_{m}.\times \left(\hat{P}\hat{Q^{T}}\right)-X_{m}^{c}{\|}^{2}$15: **if** pre_e>e and index>0 **then**16:  e_diff=pre_e−e17:  $\hat{P}\leftarrow \hat{P}$18:  $\hat{Q}\leftarrow \hat{Q}$19:  pre_e←e20: **end if**21:**end while**22:${\hat{X}}_{m}\leftarrow \hat{P}{\hat{Q}}^{T}$23:**for***i* from 1 to $R_{m}$ **do**24: **for** *j* from 1 to *T* **do**25:  $\hat{x}{}_{i,j}=\hat{x}{}_{i,j}+\Omega_{m}\cdot C_{m}$26: **end for**27:**end for**28:Output ${\hat{X}}_{m}$


## 6. Performance Evaluation

### 6.1. Setup

In this section, we implement the simulation model based on the system architecture presented in Section 3. Specifically, the simulation model is built on the realistic taxis traces from 8 am to 8 pm, 1 and 2 October 2016, in Chendu City, Sichuan Province, China, which is downloaded from Didi Chuxing GAIA Initiative accessed on [32]. The format of traffic data item is shown in Table 1. The speed of the vehicle is computed by the longitude and altitude of two adjacent sampled traffic data item.

**Figure 5:**
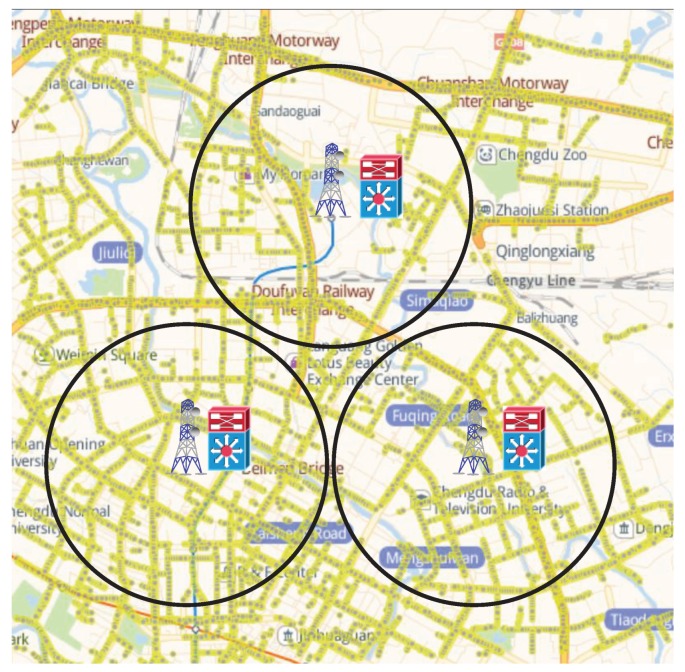
Simulation model.

The data set of the first day is used for evaluating the priority of road segments and the data of the second day is used for traffic data collection and evaluation. In statistics, the total number of taxis is 30,000. The sampling rate is [0.25,0.5] per second, which indicates that the vehicle periodically requests for data upload in every 2∼4 s. The simulation area is the core district within the second ring road of Chengdu City, as shown in Figure 5. The mapping method from the traffic data to the road segment in the real-world map is based on the literature [33]. The whole area is divided into three sub-areas and each sub-area is monitored by a MEC server, which manages around 60∼80 road segments. Furthermore, the communication capacity of each MEC server is defined as the maximum number of traffic data item uploaded by vehicles per time unit, whose default value is set to [2600,3080] data items per minute.

For performance comparison, we implement one data collection strategy and one data recovery strategy. The data collection strategy is the data random collection (DRC) strategy, which randomly selects $S^{c}\left(i,j\right)$ from $S^{o}\left(i,j\right)$ within the constraint of limited wireless bandwidth. The data recovery algorithm is K-Nearest Neighbor (KNN) with Mean [34]. Accordingly, we implement two competitive algorithms, DRC + CDR and BDC + KNN with MEAN, respectively. Besides, for performance evaluation, we define the average estimation error (AEE) to evaluate the magnitude of estimation error, which is defined in Equation (17). Specifically, high value of AEE represents that the difference between estimated traffic state and the realistic one is higher, which indicates that the algorithm achieves low estimation accuracy.

(17) $$AEE=\frac{\sum_{i=1}^{R_{m}}\sum_{j=1}^{T}\left|x_{i,j}^{o}-{\hat{x}}_{i,j}\right|}{\sum_{i=1}^{R_{m}}\sum_{j=1}^{T}x_{i,j}^{o}}$$

### 6.2. Simulation Results

#### 6.2.1. Effect of Parameters

In this section, in order to acquire the best system performance, we test the effect of three critical parameters related to the proposed algorithm, which are regularization coefficient λ, rank factor *r* and learning rate lr, respectively. The initial values of λ, *r*, and lr are set to 0.02, 100, and 0.005. When we test one of the parameters, the other two are set to the initial values.

**Figure 6:**
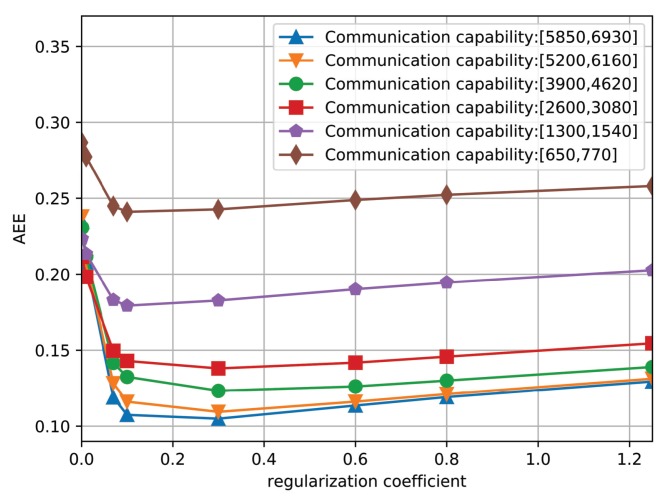
The AEE of the proposed algorithm under different regularization coefficients.

First, we conduct the performance evaluation of the proposed algorithm under different regularization coefficients, which changes from 0.001 to 1.25. In particular, Figure 6 shows the AEE curves of the proposed algorithm under six different communication capacities. It is observed that the shape of the six curves are similar and the AEE of the six curves decrease at first and then increases slowly, which indicates that there exists a point with the minimum value of AEE. Therefore, we set the value of λ to 0.07.

**Figure 7:**
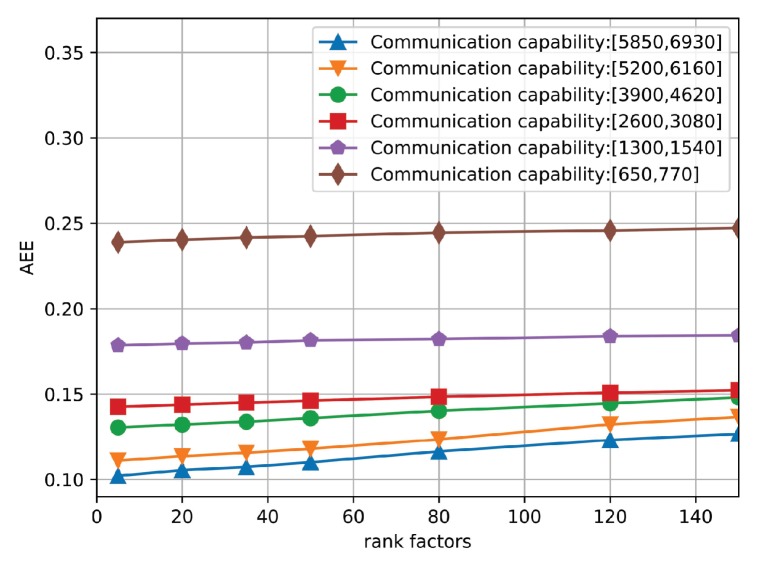
The AEE of the proposed algorithm under different rank factors.

Second, we conduct the performance evaluation of the proposed algorithm under different rank factors, which changes from 1 to 150. In particular, Figure 7 shows the AEE curves of the proposed algorithm under six different communication capacities. It is observed that six curves maintain a straight line, which indicates that the proposed algorithm is not sensitive to the rank factors. Therefore, in the simulation, we set the value of rank factor to 100.

**Figure 8:**
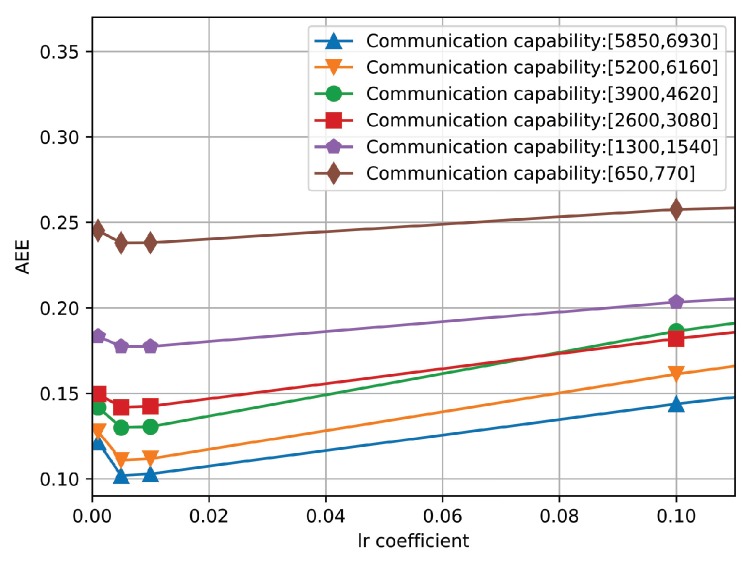
The AEE of the proposed algorithm under different learning rates.

Thirdly, we conduct the performance evaluation of the proposed algorithm under different learning rates, which changes from 0.001 to 0.1. In particular, Figure 8 shows the AEE curves of the proposed algorithm under six different communication capacities. It is observed that the AEE of the proposed algorithms first decreases and then increases slowly. In particular, the minimum value of lr is achieved when lr achieves 0.005. Therefore, in the simulation, we set the value of lr to 0.005.

#### 6.2.2. Effect of Communication Capacity

**Figure 9:**
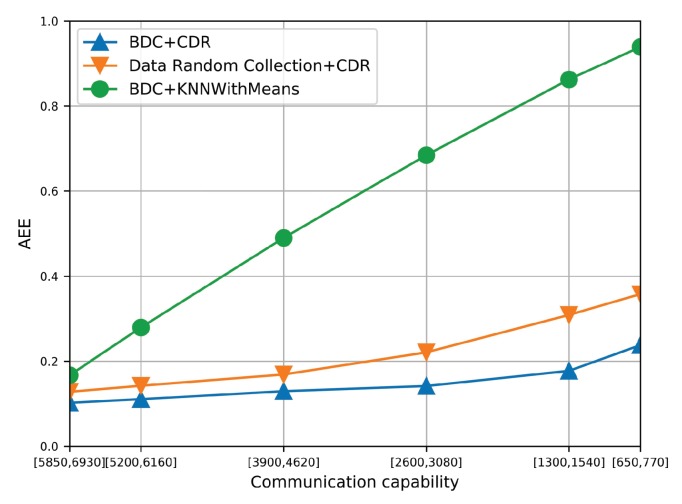
The AEE of three algorithms under different communication capacities.

Figure 9 shows the AEE of the three algorithms under different communication capacities. With decreasing communication capacity, the AEE of the three algorithms increases. It is because lower communication capacity brings lower amount of collected data, which provides less useful information for traffic recovery. Furthermore, the AEE of BDC + CDR is lower than that of RDC + CDR in all cases, which validates the effectiveness of BDC. Then, the BDC + CDR also achieves lower AEE than BDC + KNN with Mean, which indicates that the CDR can achieve higher estimation accuracy under the same data collection strategy.

**Figure 10:**
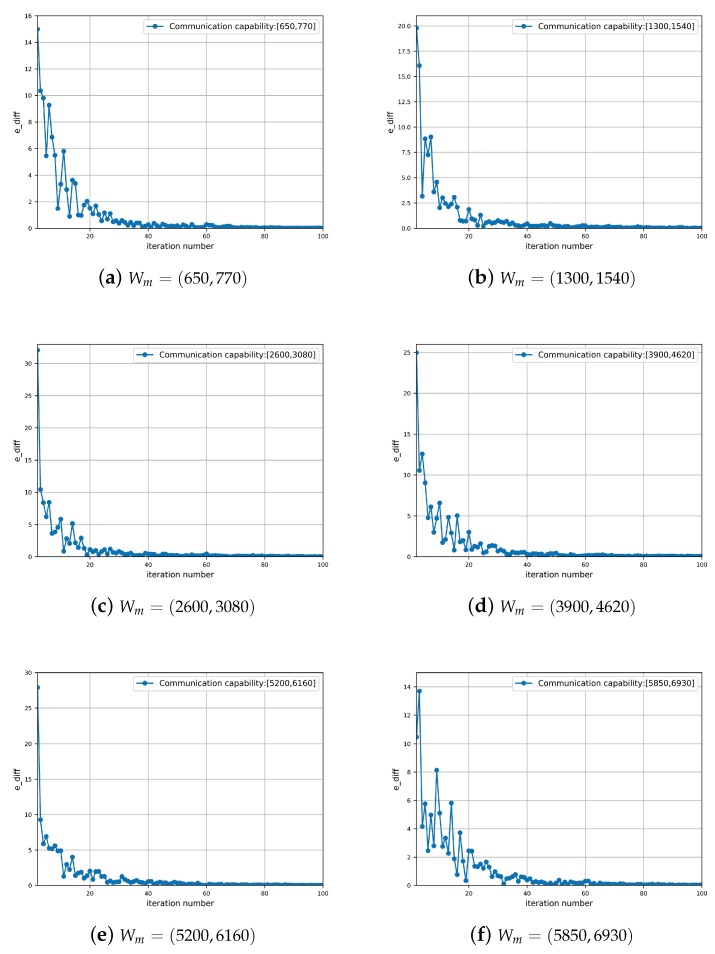
The e_diff of the proposed algorithm under different communication capacities.

Furthermore, to validate the convergence efficiency of the CDR, Figure 10 compares the e_diff under different communication capacities. In particular, e_diff is defined as the difference of *e* (shown in line 14 in Algorithm 2) between two adjacent iterations. It is observed that the e_diff converges to the zeros point efficiently across all the cases. In particular, the stop criterion is satisfied before the iteration number achieves 60, which indicates that the maximum iteration number can be considered to be a small constant. Therefore, this set of simulation results validates the efficiency and superiority of the proposed algorithm against heterogeneous communication capacities.

#### 6.2.3. Effect of Covered Road Segment Number

**Figure 11:**
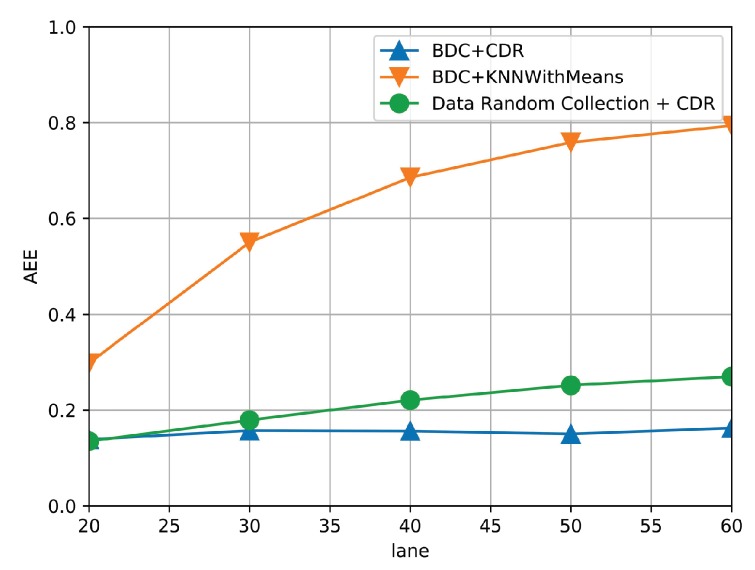
The AEE of three algorithms under different numbers of covered road segments.

Figure 11 shows the AEE of the three algorithms under different numbers of covered road segments. Specifically, the number of covered road segments is determined by the service range of MEC server. In this set of simulation, the communication capacity of each MEC server is fixed to 1600 uploaded traffic data items per minute. It is observed that the AEE of three algorithms increases gradually with the increasing number of covered road segments. It is because the required amount of traffic data has exceeded the communication capacity of the MEC server. However, the BDC + CDR achieves much lower AEE than BDC + KNN with Mean, which indicates that the BDC + CDR still can find out better correlation between the missing data and collected traffic data. Additionally, the performance gap between BDC + CDR and DRC + CDR becomes larger with increasing number of covered road segments, which indicates that the proposed data collection strategy can play a critical role when the communication capacity becomes the bottleneck.

**Figure 12:**
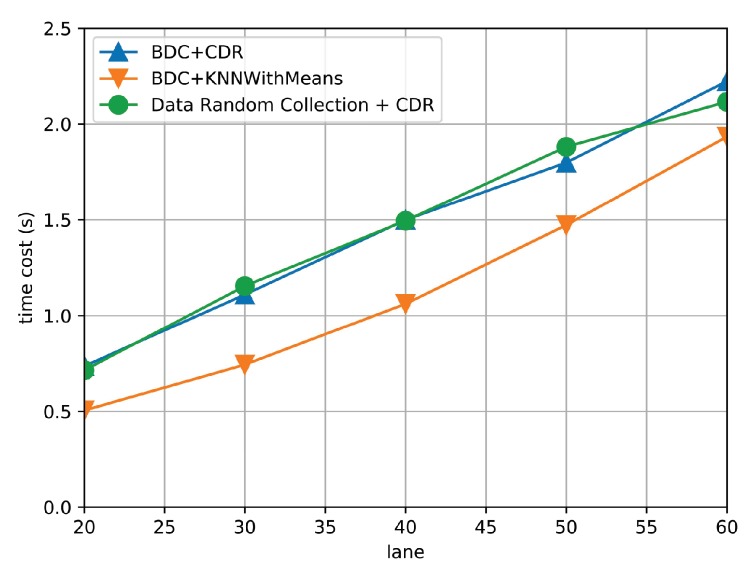
The time cost of three algorithms under different numbers of covered road segments.

Furthermore, to test the algorithm efficiency, Figure 12 compares the time cost of the three algorithms under different numbers of covered road segments. It is observed that the time cost of three algorithms increases with increasing the number of covered road segments. In particular, the time cost of proposed algorithm is almost the same to Random select + CDR, which verifies the efficiency of the BDC algorithm. Furthermore, the BDC + KNN achieves the least time cost. It is because the KNN estimates the missing traffic state by only averaging the traffic state of road segments with small cosine similarity but the CDR is based on measured traffic state matrix. However, the gap between the BDC + KNN and the proposed algorithm is small, and the proposed algorithm achieves much lower AEE than BDC + KNN. Additionally, the time cost of the proposed algorithm is linear to the number of covered road segments, which validates the time complexity analysis of Algorithm 2 in Section 5. Therefore, this set of simulation results show the adaptiveness of the proposed algorithm against different scales of traffic sensing areas.

#### 6.2.4. Effect of MEC-Based and Centralized Architectures

**Figure 13:**
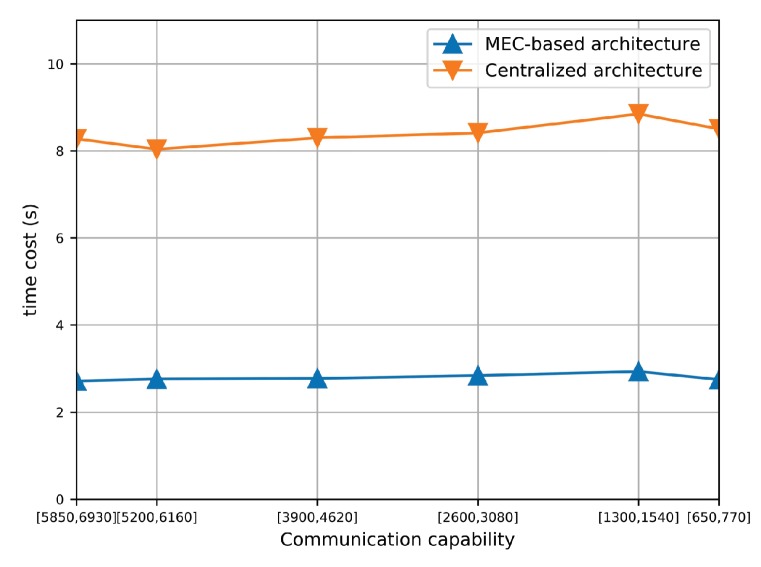
Time cost of the proposed algorithm under MEC-based and centralized architectures.

Additionally, we also compare the performance of the proposed algorithm under two types of architectures, MEC-based and centralized architectures. As shown in Table 2, The AEE of the proposed algorithm under two architectures maintains almost at the same level, which indicates that the proposed algorithm can perform well against different scales of traffic evaluation matrix. Furthermore, Figure 13 shows the time cost of the proposed algorithm under both MEC-based and centralized architectures. It is observed that the time cost of the MEC-based architecture is much smaller than the centralized architecture. It is because that in MEC-based architecture, the traffic estimation problem is decomposed into multiple subproblems and solved in parallel way, which greatly reduces the required computation resources. This set of simulation results validates the scalability of the proposed algorithm as well as the advantage of the MEC-based architecture.

#### 6.2.5. Effect of Additive Noise

**Figure 14:**
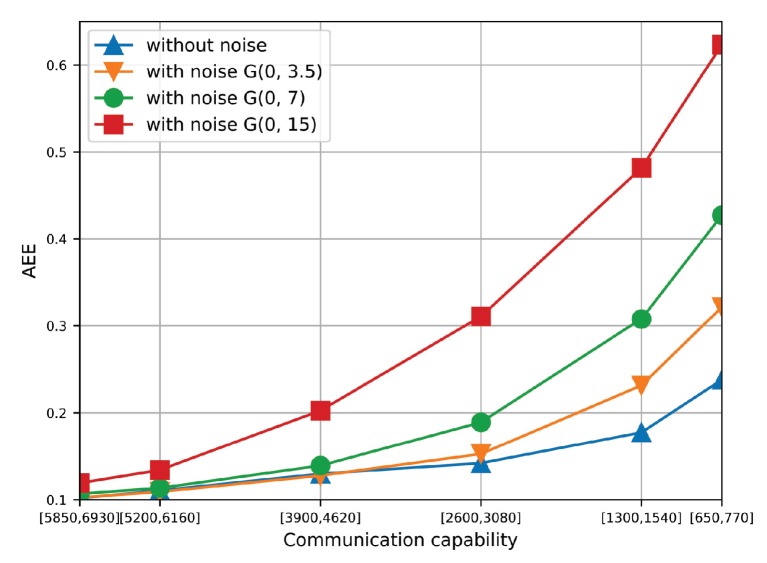
The AEE of the proposed algorithm under different magnitudes of additive noise.

Figure 14 compares the AEE of the proposed algorithm under different magnitudes of additive noise. Specifically, we add the white Gaussian noise to the original data set and perform traffic state estimation based on the contaminated data. The white Gaussian noise is represented by G(u,δ), where *u* and δ is the mean and standard deviation. As the average speed of vehicles is 35 km/h in statistics, G(0,3.5), G(0,7) and G(0,15) represents 10%, 20% and 40% noise added to the original data, respectively. As shown in Figure 14, when the communication capacity is high, white Gaussian noise does not affect the AEE too much even with 40% noise. When the communication capacity reduces, white Gaussian noise can increase the AEE greatly and results in low accuracy of traffic estimation. Therefore, it is necessary to add some denoising approach [35] to filter the noise from the collected data set, which is caused by uncorrelated factors, such as the parking vehicle along the roadside and other outlier measurements. However, it is observed that when the magnitude of noise is less than 20%, the proposed algorithm can still work well when the communication capacity is high. Therefore, this set of simulation results shows the reliability of the proposed algorithms against noisy data on the condition of high communication capacity.

## 7. Conclusions and Future Work

This paper investigated the traffic sensing system in MEC-based vehicular networks, where each MEC server is equipped with wireless AP and computation server is responsible for collecting the traffic data of each road segment and recovering the traffic state matrix based on the collected traffic data. On this basis, we formulated the bandwidth-constrained traffic sensing (BCTS) problem by synthesizing the heterogeneous communication capacities of MEC servers and the dynamic mobility features of vehicles, which aims at minimizing the AEE. To tackle the BCTS problem, we first proposed the BDC algorithm, which selects the optimal collected data set by evaluating the priority of each road segment while satisfying bandwidth constraint. In particular, the priority function is designed based on SVD, which can effectively capture the hidden structure of collected traffic state matrix. For data recovery, we proposed the CDR algorithm to minimize estimation error by transforming the BCTS into an $l_{2}$-norm minimization problem. A gradient descend method is proposed to derive the solution in an efficient way. Last but not the least, we implemented the simulation model based on realistic vehicular traces and implemented the proposed algorithms as well as two competitive algorithms. The comprehensive simulation results verify the superiority of the proposed algorithm in a wide range of service scenarios.

For future work, we would like to establish a more realistic traffic sensing system by incorporating traffic state estimation of marginal road segments, which are not covered by any MEC servers. Therefore, the potential cooperation between adjacent MEC servers would be investigated to infer the traffic state of uncovered road segment based on the knowledge of neighboring road segments. Additionally, the proposed algorithm is not suitable for the rare exceptional practical cases, such as suddenly opening or closing a road section and traffic accident. Detecting, modelling, and recovering traffic state in these extreme cases needs series of extra mechanisms, which will also be incorporated in our future work.

## Figures and Tables

**Table 1:** The data format of each traffic data item

Field	Driver ID	Time stamp	Altitude	Longitude
Type	String	String	String	String
Example	jkkt	1,501,584,540	104.04392	104.04392
Description	Desensitization	Unix time stamp, second	GCJ-02 coordinate	GCJ-02 coordinate

**Table 2:** Estimation error comparison between MEC-based and centralized architectures.

Communication Capacity	[5850,6930]	[5200,6160]	[3900,4620]	[2600,3080]	[1300,1540]	[650,770]
MEC-based architecture	0.10185	0.11081	0.13002	0.14193	0.17738	0.23793
Centralized architecture	0.09587	0.10683	0.12986	0.1416	0.17181	0.23663
